# Essential Oil Derived from Horticultural By-Products of *Artemisa dracunculus* L.: A Sustainable Source of Bioactive Compounds with Multiple Biological Activities

**DOI:** 10.3390/molecules31152583

**Published:** 2026-07-24

**Authors:** Flavio Polito, Vincenzo Candido, Giovanna Potenza, Filomena Nazzaro, Florinda Fratianni, Francesca Coppola, Vincenzo De Feo, Donato Castronuovo

**Affiliations:** 1 Department of Pharmacy, University of Salerno, Via Giovanni Paolo II, 132, 84084 Fisciano, Italy; fpolito@unisa.it (F.P.); defeo@unisa.it (V.D.F.); 2 Department of Agricultural, Forestry, Food and Environmental Sciences, University of Basilicata, Via dell’Ateneo Lucano, 10, 85100 Potenza, Italy; giovanna.potenza@unibas.it; 3 Institute of Food Sciences, CNR-ISA, Via Roma, 64, 83100 Avellino, Italy; filomena.nazzaro@isa.cnr.it (F.N.); florinda.fratianni@isa.cnr.it (F.F.); 4 Department of Agricultural Sciences, University of Naples Federico II, Via Università, 80055 Portici, Italy; francesca.coppola2@unina.it

**Keywords:** tarragon by-products, essential oil, antioxidant, amylase, glucosidase, phytotoxic, antibacterial, antibiofilm

## Abstract

Agricultural and horticultural by-products represent an underexploited source of valuable bioactive compounds that can contribute to the development of more sustainable production systems. This study investigated the chemical composition and biological activities of the essential oil obtained from the horticultural byproducts of *Artemisia dracunculus*. The waste aerial biomass was subjected to steam distillation, and the essential oil was characterized by gas chromatography–mass spectrometry. Its antioxidant, α-amylase and α-glucosidase inhibitory, phytotoxic, antibacterial, and antibiofilm activities were subsequently evaluated. The essential oil was characterized as an estragole-rich chemotype (70.17%), with *trans*-β-ocimene and *cis*-β-ocimene as other main constituents. The essential oil showed moderate antioxidant activity and measurable enzyme inhibitory activity and, despite showing limited effects on seed germination, it significantly inhibited radical elongation in selected plant species. Furthermore, it demonstrated excellent antibiofilm activity, significantly reducing the metabolic activity of mature cells of bacteria associated with biofilm formation: *Listeria monocytogenes*, *Pseudomonas aeruginosa*, *Escherichia coli*, *Acinetobacter baumannii*, *Klebsiella pneumoniae* and *Staphylococcus aureus*. The results demonstrate how horticultural by-products from *A. dracunculus* maintain a chemical profile comparable to conventional plant material and represent a valuable source of bioactive compounds with promising potential for sustainable agri-food applications.

## 1. Introduction

Among aromatic species of agricultural and food interest, *Artemisia dracunculus* L. represents a particularly interesting and still little-explored case. Commonly known as tarragon, this plant belongs to the genus *Artemisia*, one of the largest and most widespread genera of dicotyledonous angiosperms [1] which includes over 500 species, distributed mainly in the temperate zones of Europe, Asia, and North America [2]. *A. dracunculus* is native to Siberia and Mongolia [3] and is currently present in Central Asia, Mediterranean region, Eastern Europe, and North America [4]. In Europe, this species is a widespread crop, mainly used as an aromatic and edible herb for its characteristic aniseed aroma and flavor [4]. It is widely used in sauces, such as béarnaise, in meat, fish, and egg dishes, and to flavor vinegars and traditional beverages in Russia and the Caucasus region [5]. It is also used in Italy as a spice to flavor various dishes, and in some areas of the Northeast, such as Sappada (Friuli-Venezia Giulia region), it is a traditional ingredient in the production of the local “saurnschotte” cheese [6]. In addition to its use as a food, *A. dracunculus* is traditionally used in several Asian countries for the treatment of digestive disorders and other ailments, finding wide use in folk medicine [3,5,7,8,9,10,11]. Subsequent studies have confirmed numerous biological properties of the species and its metabolites, including antibacterial and antifungal [12,13], anti-inflammatory and analgesic [14], antioxidant [15,16], immunomodulatory [17,18], hepatoprotective [16], hypoglycemic [19] and anticancer [20] activities. Interest in *A. dracunculus* is mainly related to its essential oil (EO), characterized by the presence of methylcavicol (estragole) as the predominant component and numerous other compounds belonging to the class of monoterpenes, sesquiterpenes, and phenylpropanoids [3,4,5,21,22].

Due to its complex phytochemical profile, the EO of *A. dracunculus* has attracted growing interest for its potential biological applications. However, despite the available evidence, several of its potential biological properties remain poorly explored. At the same time, a still little-considered aspect concerns the possibility of obtaining this EO from secondary matrices and by-products of the agri-food chain. The development and affirmation of circular economy principles have in recent years promoted the need to valorize waste and by-products from the agri-food industry as a sustainable source of high-value bioactive compounds [23,24]. In the aromatic herbs supply chain, for example, the harvesting, selection, and packaging phases produce non-marketable fractions consisting of damaged or discarded plant material that does not comply with sales standards [25]. These residues, generally destined for disposal and considered of low commercial value, may, however, still contain secondary metabolites of interest and therefore represent a resource for the recovery of high-value products [26,27]. In this context, the use of EOs represents a particularly interesting strategy, as they constitute one of the main forms of chemical interaction between plants and the environment and can be recovered even from plant material considered waste [23,24,28]. Their natural origin, combined with their biodegradability and reduced environmental persistence, makes them promising candidates for sustainable applications in various fields [29]. However, considering the case of *A. dracunculus*, alongside this potential application, a twofold gap is evident: on one hand is the limited knowledge of the biological activities of its EO outside the most studied contexts; on the other, the almost total absence of studies focused on EOs obtained from its processing waste.

In light of these considerations, this study was conceived with a dual objective: first, to verify whether the EO obtained from *A. dracunculus* waste has a chemical profile comparable to that obtained from conventional plant material; second, to evaluate its biological potential through an integrated approach including antioxidant, enzyme inhibitory, phytotoxic, antibacterial, and antibiofilm activities. These investigations are relevant for both crop protections and food safety applications, particularly in relation to natural alternatives to synthetic agrochemicals and antimicrobial preservatives. However, the high levels of estragole raise safety concerns. Estragole has demonstrated genotoxic and carcinogenic potential in experimental animal models, and for this reason, regulatory authorities recommend minimizing human exposure, particularly in products intended for food use [30,31]. However, the risk associated with EOs containing estragole in large quantities depends on various factors such as dose, route and frequency of exposure, as well as the overall chemical composition of the EO, which may modulate its biological effects [32,33].

Thus, the work aims not only to expand knowledge on the biological activities of *A. dracunculus* EO but also to highlight how the recovery of waste matrices can represent a concrete and unconventional resource for obtaining bioactive compounds, thus contributing to their valorization within the framework of the circular economy. Finally, the data generated could help in a more comprehensive evaluation of both the efficacy and safety of this EO in potential agri-food applications.

## 2. Results

### 2.1. Yield and Chemical Composition of the Essential Oil

**Figure 1:**
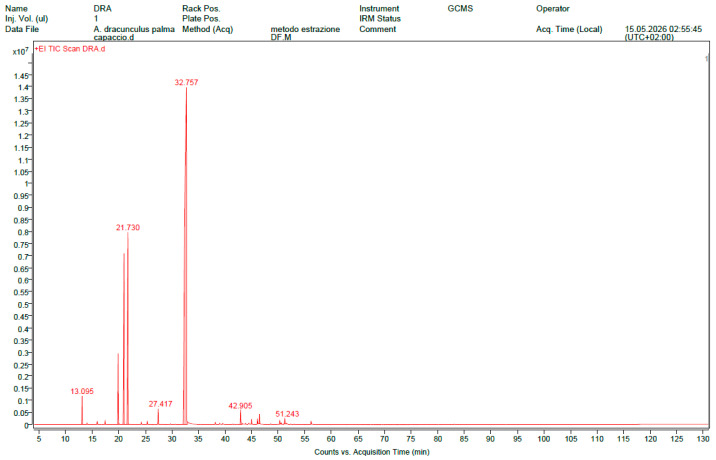
GC-MS chromatogram of *A. dracunculus* EO.

**Figure 2:**
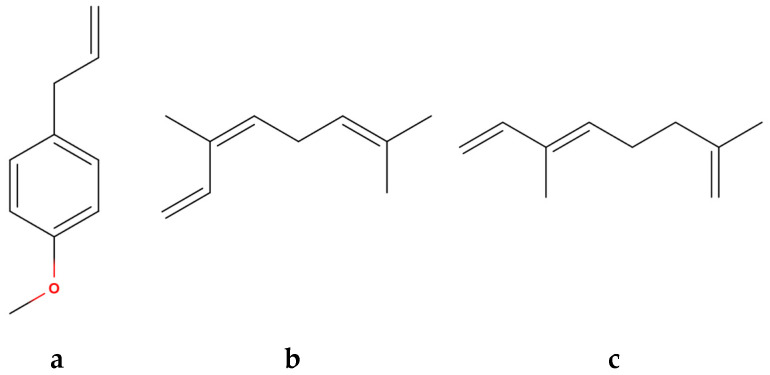
(**a**) Estragole, (**b**) *cis*-β-ocimene, (**c**) *trans*-β-ocimene.

The steam distillation of 2188.30 g of fresh plant material produced 10.23 g of EO, with a yield, calculated on fresh weight, of 0.47%. The composition of the EO is shown in Table 1, where the components are listed according to their elution order on a HP-5MS column. The GC-MS analysis led to the identification of sixteen compounds, representing 100% of the total EO. Phenylpropanoids were the predominant class (71.27%), followed by monoterpene hydrocarbons (27.42%). Estragole was the major constituent (70.17%), followed by *trans*-β-ocimene (12.27%) and *cis*-β-ocimene (9.89%). Full chromatogram is reported in Figure 1 and the structural formulas of the main components of EO are reported in Figure 2.

### 2.2. Antioxidant Activity

The EO showed antioxidant activity in all three assays performed, as reported in Table 2. The antioxidant capacity was moderate, with higher values observed in the FRAP and ABTS assays. However, the antioxidant power of the EO was consistently lower than that observed for the reference compounds (trolox for DPPH and FRAP and ascorbic acid for ABTS).

### 2.3. Inhibitory Activity of α-Amylase and α-Glucosidase

The EO inhibited both enzymes, showing measurable effects as reported in Table 3. The activity was more pronounced against α-amylase, as indicated by the lower IC_50_ value. However, the inhibitory potency was considerably lower than that of the reference substance used, acarbose, which showed lower IC_50_ values.

### 2.4. Phytotoxic Activity

Table 4 shows the phytotoxic activity of EO on the germination and radical elongation of four selected seeds: the two crops, *H. vulgare* and *R. sativus* and the two weeds *L. multiflorum* and *S. alba*. The table shows the results as percentage inhibition (%) compared to the treatment carried out with the control solution consisting of water and acetone (99.5-0.5 *v*/*v*), which was assigned an inhibition of 0.0%. In the table, the results were presented using green for positive inhibitions and red for negative inhibitions (i.e., a process-stimulating effect). White was used when the activity was 0.0%. The more intense the color, the greater the activity.

**Figure 3:**
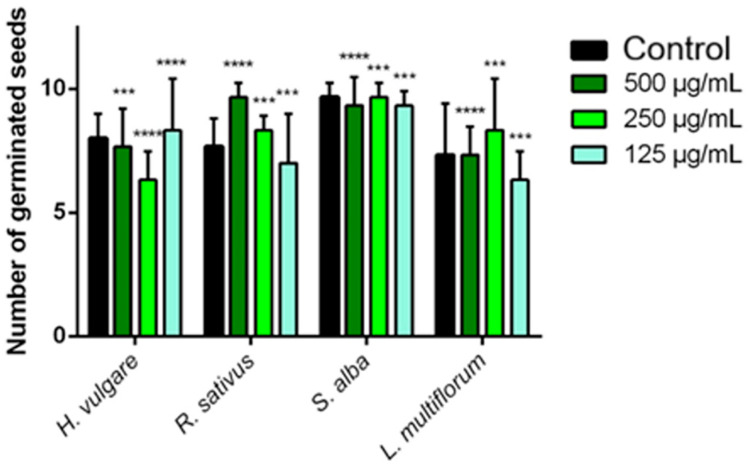
Phytotoxic activity of the EO against the germination of *H. vulgare*, *R. sativus*, *S. alba* and *L. multiflorum*. The results are reported as the mean of three experiments ± the standard deviation *** *p* < 0.001; **** *p* < 0.00001 compared with control (ANOVA followed by Dunnet’s multiple comparison test).

**Figure 4:**
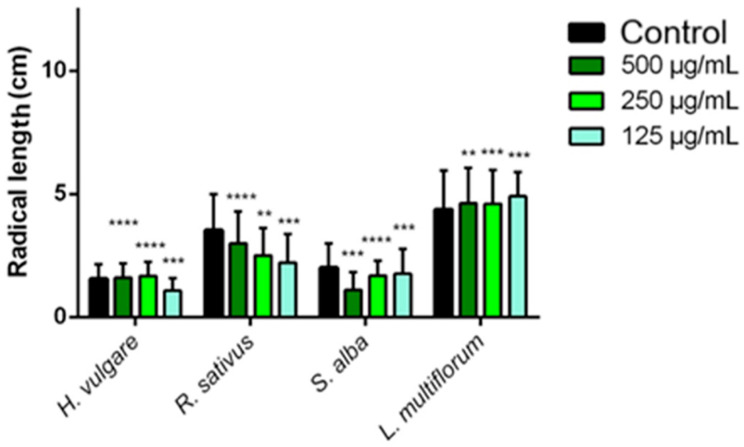
Phytotoxic activity of the EO against root elongation of *H. vulgare*, *R. sativus*, *S. alba* and *L. multiflorum*. The results are reported as the mean of three experiments ± the standard deviation ** *p* < 0.01; *** *p* < 0.001; **** *p* < 0.00001 compared with control (ANOVA followed by Dunnet’s multiple comparison test).

Figure 3 and Figure 4 show bar graphs used to describe the same effects of the EO solutions on the germination and radical elongation processes of the seeds. The data were constructed using measurements taken directly on the seeds grown: number of germinated seeds and cm of root length.

The EO is poorly effective in inhibiting the germination of all the seeds considered. The greatest inhibitory activity was recorded against *H. vulgare*, at a 250 µg/mL (20.88%) and *L. multiflorum* at 125 µg/mL (13.64%). In all other cases the inhibition does not exceed 10% and in many others the EO even promotes the germination processes. The EO was found to be more active in inhibiting root elongation. The greatest inhibitory activity was against *S. alba* and *R. sativus* at all concentrations tested. In the case of *R. sativus*, the inhibition varies from 14.73% at 500 µg/mL to 37.39% at 125 µg/mL. In the case of *S. alba*, the inhibition ranged from 11.50% (125 µg/mL) to 44.50% (500 µg/mL). In the other cases, the EO promoted an increase in radical elongation for *L. multiflorum*, while for *H. vulgare* the EO poorly inhibited the radical elongation at 500 µg/mL (3.23%), promoted radical elongation at 250 µg/mL (−7.74%) and caused an inhibition at 125 µg/mL (30.32%).

### 2.5. Antibiofilm Activity

The antibiofilm activity of the EO was evaluated against 24 h preformed biofilms of *Acinetobacter baumannii*, *Escherichia coli*, *Klebsiella pneumoniae*, *Listeria monocytogenes*, *Pseudomonas aeruginosa*, and *Staphylococcus aureus* using CV and MTT assays at concentrations of 2, 4, and 6 µL/mL (Table 5). The tested concentrations were selected based on the MIC values, which were relatively homogeneous among the six pathogens, ranging from 10 to 12 µL/mL: 10 ± 1 μL/mL for *A. baumannii*, *E. coli* and *S. aureus*, 11 ± 1 μL/mL for *K. Pneumoniae* and *L. monocytogenes*, and 12 ± 1 μL/mL for *P. aeruginosa*. The MTT assay showed an evident concentration-dependent reduction in sessile-cell metabolic activity. Notably, measurable inhibitory effects were already observed at the lowest tested concentration (2 µL/mL), corresponding to approximately one-fifth of the MIC values. At this concentration, metabolic inhibition ranged from 10.12% in *P. aeruginosa* to 40.00% in *L. monocytogenes*. Increasing the EO concentration to 4 µL/mL significantly enhanced the activity, with inhibition values ranging from 29.16% (*S. aureus*) to 61.42% (*L. monocytogenes*). The strongest effects were observed at 6 µL/mL, where metabolic inhibition reached 55.23, 75.79, 69.28, 79.91, 75.40, and 74.80% against *A. baumannii*, *E. coli*, *K. pneumoniae*, *L. monocytogenes*, *P. aeruginosa*, and *S. aureus*, respectively. The CV assay revealed a more moderate but still concentration-dependent effect on total biofilm biomass. At 2 µL/mL, biomass reduction was evident for most tested strains, exceeding 20% for *A. baumannii*, *K. pneumoniae*, *P. aeruginosa*, and *S. aureus*. At the highest concentration tested (6 µL/mL), biomass reduction reached 45.00% for *A. baumannii*, 34.47% for *E. coli*, 22.56% for *K. pneumoniae*, 29.39% for *L. monocytogenes*, 51.25% for *P. aeruginosa*, and 41.04% for *S. aureus*. Overall, the EO exhibited a substantially stronger effect on the metabolic activity of sessile cells than on biofilm biomass removal (Table 6). According to the MTT assay, *L. monocytogenes*, *P. aeruginosa*, *E. coli*, and *S. aureus* were the most susceptible strains, whereas *K. pneumoniae* exhibited the lowest reduction in biofilm biomass according to the CV assay. Despite relatively similar MIC values among the tested microorganisms, marked differences were observed in their biofilm responses, suggesting that the anti-biofilm activity of the EO is influenced by biofilm-specific traits rather than by planktonic susceptibility alone.

## 3. Discussion

The yield of *A. dracunculus* EO depends on environmental and phenological differences. Recent studies show that geographic variability (climate, soil, and pedoclimatic conditions) can significantly influence the biosynthesis of volatile metabolites, and therefore the percentage yield [4,34]. Likewise, the plant’s developmental stage is a key factor: early vegetative growth typically results in lower yields than pre-flowering or early flowering, when terpene accumulation is generally highest [34]. In this context, the yield of 0.47% on fresh weight obtained from material grown in Capaccio in May is consistent with recent literature, which reports a range from 0.4 to 1.0% for the percentage yield and above all lower values for spring harvests and in Mediterranean conditions compared to summer ones or at full phenological maturity, where yields tend to be higher [4,5,34]. Chemical composition is also influenced by various factors, such as habitat, soil salinity, and plant age, which affect both the qualitative and quantitative profile [3]. However, it is possible to identify recurring main components in several studies. Estragole is generally indicated as the predominant compound, with amounts that can vary between 40 and 85%. Other frequently reported constituents among the main ones include elemicin (up to 57%), methyleugenol (up to 25%), terpinen-4-ol, and sabinene (up to 40%). Other recurring compounds, which reach amounts up to 20%, are terpinolene, limonene, and *cis*- and *trans*-ocimene, *trans*-anethole, α-phellandrene, β-phellandrene, and (Z)-artemidine [3,5,12,35,36,37,38,39,40,41]. The EO obtained from *A. dracucunculus* waste was characterized by a clear predominance of estragole (70.17%) and smaller amounts of *cis*- and *trans*-β-ocimene, limonene, α-pinene, and eugenol. This compositional profile is fully consistent with the estragole-rich chemotype frequently reported for *A. dracunculus* EO, in which this compound generally represents the predominant constituent and can represent over 70% of the volatile fraction [4,5,42]. Interestingly, the recovery of EO from waste matrices did not result in substantial changes in the qualitative composition, which is comparable to that described for EOs obtained from conventional plant material. Similarly, the quantitative profile also shows a high comparability with the Italian samples reported in the literature, in which estragole generally represents the main constituent (up to 73.3–82%), while the observed differences mainly concern the relative abundance of secondary metabolites. As also reported in studies conducted on samples from other geographical areas, these quantitative variations reflect the known chemotypic variability of the species, without modifying the general estragole compositional pattern [31,35,43,44,45]. This intrinsic variability, however, should be considered with a view to potential future industrial exploitation of *A. dracunculus* processing waste. Differences in the quantities of the main constituents between batches of raw material, despite maintaining the predominantly estragole chemotype, can affect the reproducibility and biological performance of EO. Therefore, the development of standardized protocols for harvesting, storing, and extracting EO from biomass, along with confirmed chemical characterization of the composition, will be essential to ensure consistent quality and efficacy.

In recent years, the growing demand for crops to satisfy the ever-expanding world population and the need to achieve ever-higher yields have led to the widespread and sometimes uncontrolled use of synthetic herbicides for weed control [46]. Despite the undoubted effectiveness of synthetic products, they are unfortunately known to be a cause of environmental pollution (water and soil contamination) and a risk to human health due to their accumulation in the body following the consumption of treated products [47]. For these reasons, and to prevent the emergence of resistance, the search for environmentally friendly and safer alternatives to these products is ongoing. One such alternative could be the use of substances often involved in the interaction of plants with the environment, such as EOs, characterized by a strong allelopathic and phytotoxic component [48,49]. The phytotoxic activity observed for *A. dracunculus* EO could result from the combined action of several mechanisms involved in seed germination and early seedling development. In particular, the inhibition of α-amylase and α-glucosidase could limit the mobilization of starch reserves necessary for embryo growth, representing one of the mechanisms potentially involved in the observed phytotoxic effects [50,51,52]. the moderate antioxidant activity detected in this study may contribute to the modulation of reactive oxygen species (ROS), during seed germination and radicle elongation [53,54] but it is unlikely to represent the main factor responsible for the observed effects. Therefore, the phytotoxic effects observed in the present study are likely the result of a multi-target mode of action rather than the modulation of a single biological process.

The limited data available in the literature show discordant results regarding the antioxidant activity of *A. dracunculus* EO. Some studies report IC_50_ values, obtained through the DPPH assay, between 0.070 [55] and 3,19 mg/mL. An EO studied by Mrbati and collaborators [56] showed instead a powerful antioxidant activity, much higher than that found in this work, for all three assays (DPPH, FRAP and ABTS), with IC_50_ values of 84.44 ± 5.98 µg/mL, 160.38 ± 8.56 µg/mL and 96.71 ± 1.52 µg/mL respectively. Such variability probably reflects differences in the chemical composition of the EO related to genotype and environmental conditions. To the best of our knowledge, however, no studies have investigated the antioxidant activity of EOs obtained from *A. dracunculus* processing waste. Likewise, no studies have evaluated the inhibitory activity of *A. dracunculus* EO against α-amylase and α-glucosidase, either from conventional plant material or processing waste. Previous investigation only considered different plant extracts, reporting heterogeneous inhibitory activities against human enzymes, with IC_50_ values lower than those in this study, ranging between 1.41 and 13.59 mg/mL for α-amylase and 0.20 and 5.45 mg/mL for α-glucosidase [57,58,59]. The activity could be attributable to the massive presence of estragole for which, although studies are still few, a certain inhibitory activity on α-amylase [37,38,39], and α-glucosidase [60,61] has been highlighted, whereas no evidence is currently available for *trans-* and *cis*-β-ocimene, the other main components found in our EO. However, since no kinetic analyses were performed, the mechanism of inhibition (e.g., competitive or non-competitive) and the specific contribution of individual EO constituents cannot be confirmed. The observed inhibition should therefore be considered as the result of the overall activity of all EO constituents, with individual components possibly contributing differently. Only one study investigated the phytotoxic properties of an EO of *A. dracunculus* [43], demonstrating a marked inhibition of the germination of *Papaver rhoeas* L. and *Avena fatua* L., as well as radicle elongation in *A. fatua*, *P. rhoeas* and *Lepidium sativum* L., with only little effects on *Raphanus sativus* L. Also, in this case, the activity could be linked to the massive presence of estragole, for which inhibitory properties have been reported in various species (*Allium cepa* L., *Lactuca sativa* L., *R. sativus* and *Lepidium sativum* [62,63,64]. Components such as *trans*- and *cis*-β-ocimene may also contribute to the phytotoxic potential of the EO, as numerous publications demonstrate their activity on various plant species [*L. sativa*, *Phalaris minor* Retz., *Triticum aestivum* L., *Cassia occidentalis* (L.) Link] [63,65,66,67].

Subsequently, considering the widespread use of *A. dracunculus* as a culinary herb, the evaluation of its antimicrobial and antibiofilm potential may be of particular interest for both food preservation and public health. EOs have attracted increasing attention as natural antimicrobial agents since they can inhibit both planktonic bacterial growth and biofilm formation through multiple mechanisms of action, thus representing promising alternatives to synthetic preservatives and complementary tools against antibiotic-resistant pathogens [68,69,70].

The anti-biofilm assays showed clear concentration-dependent activity against 24 h preformed biofilms. Notably, anti-biofilm effects were already identifiable at 2 µL/mL, a concentration corresponding to approximately 17–20% of the MIC values determined for the planktonic counterparts. At this sub-inhibitory concentration, reductions in both sessile-cell metabolism and biofilm biomass were observed for most tested microorganisms, indicating that the EO can interfere with biofilm-associated physiology even at concentrations well below those required to inhibit planktonic growth. Similar sub-MIC effects have been reported for several plant EOs and have been associated with alterations in membrane function, cellular communication, and biofilm homeostasis [71,72]. Interestingly, the EO exerted a substantially stronger effect on sessile-cell metabolic activity than on total biofilm biomass. At the highest tested concentration (6 µL/mL), metabolic inhibition exceeded 75% for *L. monocytogenes*, *E*. *coli*, *P. aeruginosa*, and *S. aureus*, whereas biomass reduction remained below 55% for all tested species. Similar differences between MTT and CV assays have been reported for several plant EOs and generally indicate that biofilm-embedded cells are more readily inactivated than the extracellular polymeric substance (EPS) matrix is removed [73,74]. The considerable reduction in metabolic activity may be associated with the lipophilic nature of estragole and the other volatile constituents of the oil. Essential oil components are known to interact with bacterial membranes, increasing permeability, disrupting proton gradients, impairing ATP synthesis, and finally reducing cellular viability [75,76]. Such mechanisms are particularly relevant in established biofilms, where complete matrix disruption is often difficult to achieve, while cellular metabolism remains vulnerable to antimicrobial stress. Among the tested microorganisms, *L. monocytogenes* exhibited the highest susceptibility, showing approximately 79.91% inhibition of sessile-cell metabolic activity at 6 µL/mL. This finding is particularly relevant considering the persistence of *Listeria* biofilms in food-processing environments and their involvement in post-processing contamination of ready-to-eat foods [77]. Likewise, the strong activity observed against *P. aeruginosa* is noteworthy, as this species is widely recognized for its highly structured biofilms and intrinsic resistance to antimicrobial agents [78]). Despite the relatively similar MIC values observed for all tested strains (10–12 µL/mL), their 24 h established biofilms responded differently to EO treatment, highlighting that susceptibility under planktonic conditions does not necessarily predict biofilm sensitivity. For example, *P. aeruginosa*, which exhibited the highest MIC value, showed one of the greatest reductions in sessile-cell metabolic activity, whereas *A. baumannii*, despite one of the lowest MIC values, was less affected in the MTT assay. These findings suggest that the antibiofilm activity of *A. dracunculus* EO is influenced not only by planktonic susceptibility but also by species-specific biofilm characteristics, including extracellular matrix composition, biofilm architecture, and the physiological heterogeneity of sessile populations.

The response of *K. pneumoniae* was particularly noteworthy. This species exhibited the lowest reduction in biofilm biomass according to the CV assay, despite a substantial decrease in sessile-cell metabolic activity detected by the MTT assay. This apparent discrepancy is consistent with the well-known ability of *K. pneumoniae* to produce abundant capsular polysaccharides and a dense extracellular polymeric substance (EPS) matrix, which can hinder the diffusion of antimicrobial compounds and increase the structural stability of the biofilm [79]. Consequently, the EO appears to impair the viability of biofilm-embedded cells more efficiently than it disrupts the extracellular matrix itself. Similar differences between CV and MTT outcomes have been reported for other plant essential oils and reflect the complementary nature of these two assays, which evaluate distinct aspects of biofilm physiology, namely total attached biomass and the metabolic activity of viable sessile cells, respectively. Overall, these findings further support the hypothesis that the antibiofilm activity of *A. dracunculus* EO depends not only on planktonic susceptibility but also on the structural and physiological characteristics of species-specific biofilms [78,80]. Although estragole is likely the principal contributor to the observed activity due to its high abundance, synergistic interactions among minor constituents such as ocimenes, limonene, α-pinene, and eugenol cannot be excluded. Numerous studies have demonstrated that the antimicrobial efficacy of EOs often arises from complex interactions among major and minor components rather than from the action of a single constituent [75,76]. Overall, these outcomes indicate that *A. dracunculus* EO is particularly effective at reducing the viability of cells embedded within established biofilms, while exerting a more limited effect on biofilm biomass removal. The EO’s ability to exert measurable anti-biofilm effects even at concentrations far below the MIC further reinforces its potential as a promising natural agent for weakening established biofilms and enhancing the susceptibility of sessile cells to subsequent antimicrobial interventions. Future studies could investigate the activity of the EO against biofilms at different developmental stages to evaluate whether the susceptibility changes during biofilm maturation.

## 4. Materials and Methods

### 4.1. Plant Material

By-products from aerial parts of *A. dracunculus* were collected in May 2026 from Aroma Domus O.P. Sc.a.r.l., an organic farm of medicinal and aromatic plants sited in Capaccio Scalo (Salerno, Southern Italy; 40°27′ N, 15°00′ E, 120 m a.s.l.). The *A. dracunculus* crop was grown in a plastic greenhouse on a previously ploughed and fertilized fine-texture soil. Planting, by using rooted cuttings, took place on March 2025 with a spacing of 40 cm in rows spaced 60 cm apart to obtain a density of 4,2 plants per m^2^. Moreover, the normal agronomic practices (irrigation, fertilization, weed, disease and pest control) of local growers were followed. The harvest was carried out by cutting the plants 30 cm above ground level. By selecting the aerial parts suitable for marketing, waste biomass was obtained, consisting of damaged or discarded plant material which was used for distillation. The plant identification was carried out by Professor Vincenzo De Feo and a voucher specimen, labelled as DF/2026/121, is stored in the herbarium of the Pharmaceutical Botany Chair at the University of Salerno.

### 4.2. Essential Oil Extraction

Fresh aerial parts were processed by steam distillation for 2 h according to procedure described in the *European Pharmacopoeia* [81]. The recovered EO obtained was solubilized in *n*-hexane (Sigma-Aldrich, Merck Life Science S.r.l., Milan, Italy), dried using anhydrous sodium sulphate and concentrated under a gentle nitrogen steam to eliminate any remaining solvent. The EO was stored in amber glass vials at +4 °C under dark conditions and protected from heat and moisture until further analysis.

### 4.3. GC and GC-MS Analysis

The chemical characterization of the EO was performed by GC-MS analysis using an Agilent 6850 Ser. II system (Santa Clara, CA, USA) equipped with an HP-5MS fused silica capillary column (30 m × 0.25 mm; 0.25 μm film thickness) and coupled to an Agilent 5973 mass spectrometer. Spectra were acquired in electron impact ionization (EI) mode at 70 V, with ion multiplier energy of 2000 V. The mass spectra were recorded in the *m*/*z* range 40–500 amu, with an acquisition rate of five scans per second. The oven thermal program included an initial isothermal phase of 40 °C lasting 5 min, followed by a temperature increase of 2 °C/min until reaching 270 °C and a subsequent isothermal hold at that temperature for 20 min. The transfer line temperature was 295 °C. To confirm the identification of the constituents, the analyses were also repeated using an HP Innowax polar column (50 m × 0.20 mm i.d.; 0.25 μm film thickness), keeping the operating parameters unchanged. Helium was used in both cases as the carrier gas with a constant flow rate of 1.0 mL/min. The identification of the compounds was carried out by comparing the Kovats retention indices (KI) with the values reported in the literature [82,83,84,85] as well as by comparing the mass spectra obtained with those of the pure compounds available in our laboratory and with those contained in the NIST 17 and Wiley 257 libraries [86]. The Kovats indices were calculated using a homologous series of n-alkanes between C10 and C35, analyzed under the same experimental conditions. The relative percentages of the individual components were determined by normalizing the areas of the chromatographic peaks, without applying response correction factors.

### 4.4. Antioxidant Assays

#### 4.4.1. DPPH Assay

The antioxidant potential of the EO was determined through the DPPH (2,2-diphenyl-1-picrylhydrazyl) (Sigma-Aldrich, Merck Life Science S.r.l., Milan, Italy) radical scavengins assay, following the protocol previously reported by Ud-Daula et al. [87]. Briefly, EO samples were prepared in methanol (Sigma-Aldrich, Merck Life Science S.r.l., Milan, Italy) at different concentrations and aliquots of each solution were mixed with 60 μM DPPH solution to reach a final volume of 1 mL. All measurements were performed in triplicate. The control consisted of the DPPH solution without EO addition, while methanol was used as the blank. After incubation for 45 min, absorbance values were recorded at 515 nm using a Multiskan GO spectrophotometer (Thermo Fisher Scientific, Vantaa, Finland). Antioxidant activity was expressed as IC_50_, defined as the concentration of EO required to achieve a 50% decrease in DPPH absorbance and values were reported as mean ± the standard deviation (SD).

#### 4.4.2. FRAP Assay

The FRAP (ferric-reducing antioxidant power assay) was carried out following the procedure described by Benzie and Strain [88]. All measurements were performed in triplicate using a 96-well microplate. Essential oil samples, preciously diluted in methanol to obtain different concentrations, were mixed with fresh prepared FRAP reagent to a final reaction volume of 272 μL per well. The reaction mixtures were incubated in a dark environment at 37 °C for 30 min. The absorbance of the FRAP blank alone was subtracted from the absorbance of the FRAP with the samples. The results were the means of three experiments ± SD and expressed as μmol of Fe^2+^ equivalents/g of EO. Trolox was used as reference compound.

#### 4.4.3. ABTS Assay

The 2,2-azino-bis 3-ethylbenzothiazoline-6-sulfonic acid (ABTS) (Sigma-Aldrich, Merck Life Science S.r.l., Milan, Italy) assay was carried out according to the protocol described by Ud-Daula et al. [87]. All determinations were carried out in triplicate using a microplate format. Ten μL of the different sample concentrations previously dissolved in methanol to obtain different concentrations and 190 μL of ABTS were added to the wells for analysis. Control wells contained 10 μL of PBS and 190 μL of ultrapure water. Antioxidant activity was expressed as μmol of Trolox equivalent (TE) per g of EO and the results were reported as means of three experiments ± SD. Ascorbic acid was used as a reference compound.

### 4.5. α-Amylase Inhibition Assay

Jaradat’s approach, with minor modifications, was used to assess the amylase activity [89]. One hundred μL of EO solutions, previously prepared in methanol at different concentrations, were incubated with 200 μL of 20 mM sodium phosphate buffer (pH 6.9) and 100 μL of α-amylase solution (10 U/mL) (Sigma-Aldrich, Merck Life Science S.r.l., Milan, Italy) at 37 °C for 10 min. Subsequently, 180 μL of a 1% starch substrate solution was added, and the reaction mixture was further incubated at 37 °C for 20 min. The enzymatic reaction was stopped by adding 180 μL of 96 mM 3,5-dinitrosalicylic acid (DNSA) (Sigma-Aldrich, Merck Life Science S.r.l., Milan, Italy) solution, followed by heating at 100 °C for 10 min using a dry heating block. Absorbance was then measured at 540 nm with a UV spectrophotometer (Thermo Fisher Scientific, Vantaa, Finland). All tests were performed in triplicate, and inhibitory activity was expressed as IC_50_ values reported as mean ± standard deviation (SD). α-Amylase enzyme was purchased from Sigma-Aldrich (Merck Life Science S.r.l., Milan, Italy).

### 4.6. α-Glucosidase Inhibition Assay

The inhibitory activity of α-glucosidase was assessed according to the method described by Nguyen et al. [90], with slight modifications. The assays were performed in microtiter plate format by combining 150 μL of 0.1 M phosphate buffer (pH 7.0), 10 μL of EO solutions prepared in methanol at different concentrations, and 15 μL of α-glucosidase solution (1 U/mL) (Sigma-Aldrich, Merck Life Science S.r.l., Milan, Italy) in each well. After pre-incubation at 37 °C for 5 min, the enzymatic reaction was initiated by adding 75 μL of 2.0 mM 4-nitrophenyl α-D-glucopyranoside as substrate, followed by further incubation at 37 °C for 10 min. Absorbance was measured at 405 nm using a UV spectrophotometer (Thermo Fisher Scientific, Vantaa, Finland). Acarbose was used as a positive control, while phosphate buffer instead of EO solution was used as a negative control. Enzyme inhibition was calculated and expressed as IC_50_ values reported as mean ± standard deviation (SD). α-Glucosidase enzyme was obtained from Sigma-Aldrich (Merck Life Science S.r.l., Milan, Italy).

### 4.7. Phytotoxicity Assay

Germination and root elongation of seeds of two plants of agricultural interest, *Raphanus sativus* L. (radish) and *Hordeum vulgare* L. (barley), as well as two weeds, *Lolium multiflorum* Lam. (Italian ryegrass) and *Sinapis alba* L. (wild mustard) were examined to assess phytotoxic activity of the EO. Seeds commonly used in phytotoxic assessments due to their ease germination and well documented histological characteristics were used: *R. sativus* and *H. vulgare* seeds were purchased from Blumen group s.r.l., Bologna, Italy, *L. multiflorum* seeds were obtained from Fratelli Ingegnoli s.p.a., Milan, Italy. Seeds of *S. alba* were instead collected from wild populations, in June 2025, in a field located in the municipality of Fisciano (SA) coordinates 40°46′ N, 14°47′ E. *Sinapis alba* identification was carried out by Professor Vincenzo De Feo and a voucher specimen, labelled as DF/2025/78, was stored in the herbarium of the Pharmaceutical Botany Chair at the University of Salerno. After having sterilized them with 95% ethanol (Carlo Erba Reagents S.r.l., Milan, Italy) for 15 s, they were placed in Petri dishes (Ø 90 mm) on three layers of Whatman filter paper, soaked in distilled water (7 mL, for the control group) or in a solution containing the EO (7 mL) at various concentrations. Germination conditions were maintained at 20 ± 1 °C, under a 16 h light and 8 h dark photoperiod. To improve solubility, the EO was dissolved in a mixture of water and acetone (99.5:0.5 *v*/*v*) and tested at concentrations of 500, 250 and 125 μg/mL. These concentrations were selected to cover a concentration range commonly used for the biological evaluation of Eos and routinely used in the laboratory where the tests were carried out, in order to allow comparison even between different samples. No differences were observed between the control groups treated with the water–acetone mixture and those treated with water alone. The progress of seed germination was monitored in Petri dishes at 24 h intervals, with a seed considered germinated when radicle protrusion became visible [91]. After 120 h for *R. sativus*, *S. alba* and *H. vulgare* and 168 h for *L. multiflorum*, germination was observed and radicle lengths were measured in cm. Each measurement was performed in triplicate, using Petri dishes containing 10 seeds each.

### 4.8. Antimicrobial Activity

#### 4.8.1. Microorganisms and Culture Conditions

The strains used were provided by the Leibniz Institute, DSMZ-German Collection of Microorganisms and Cell Cultures GmbH, Braunschweig, Germany: *Acinetobacter baumannii* ATCC 19606, *Pseudomonas aeruginosa* DSM 50079, *Escherichia coli* DSM 8579, and *Klebsiella pneumoniae*, clinical isolate (Gram-negative), *Staphylococcus aureus* subsp. *aureus* Rosenbach ATCC 25923 and *Listeria monocytogenes* ATCC 7644 (Gram-positive). Before analysis, the bacteria were cultivated in Luria–Bertani broth at 37 °C for 18 h. *A. baumannii* was instead grown at 35 °C under the same conditions.

#### 4.8.2. Minimal Inhibitory Concentration (MIC)

*A. dracunculus* EO and Dimethyl Sulfoxide (DMSO) were subjected to ultrafiltration before use in the experiments in this study. The Minimum Inhibitory Concentration (MIC) of the EO was determined using the resazurin-based microdilution method by Sarker et al. [92], and Khedri et al. [93]. A resazurin stock solution (Sigma-Aldrich, Merck Life Science S.r.l., Milan, Italy) was prepared by dissolving 270 mg in 40 mL of sterilized deionized water. The assay was carried out in sterile 96-well microtiter plates. The first row was filled with 100 µL of samples in DMSO (1:100 *v*/*v*) and the remaining wells were filled with 50 µL of Luria–Bertani broth (Sigma-Aldrich, Merck Life Science S.r.l., Milan, Italy) or a standard sterile solution. Serial dilutions of the EO were performed in descending concentrations. This was followed by adding 10 µL of resazurin indicator solution to each well. Furthermore, 30 µL of 3.3× sensitized broth and 10 µL of bacterial suspension (5  ×  10^6^ CFU/mL) were added to each well. The plates were sealed with Parafilm to minimize evaporation and incubated at 37 °C for 24 h, except for *A. baumannii*, which was incubated at 35 °C. Tetracycline, dissolved in DMSO, was included as a positive control, while wells containing Luria–Bertani broth, resazurin, and the bacterial suspension without essential oil served as a negative control. Bacterial growth was assessed visually by monitoring the color of the resazurin indicator: a change from blue–purple to pink or colorless indicated microbial growth. The MIC was defined as the lowest concentration of essential oil that completely prevented this color change.

#### 4.8.3. Biofilm Inhibitory Activity

The effect of the EO on 24 h preformed biofilm was evaluated using sterile flat-bottom 96-well microplates following the procedure described by Khedri et al. [93]. Bacterial cultures were standardized to 0.5 McFarland using fresh culture broth. Each well of the microtiter plate was inoculated with 10 µL of the bacterial culture and incubated for 24 h at 37 °C (35 °C for *A. baumannii*). Following the removal of the planktonic cells, 2.5 or 5 µL of EO, previously dissolved in sterile DMSO (Sigma-Aldrich, Merck Life Science S.r.l., Milan, Italy), were added to each well to achieve a final EO concentration of 5–10 µg/mL. The final volume in each well was adjusted to 250 µL with Luria–Bertani broth. The plates were sealed with parafilm and incubated for an additional 24 h at 37 °C (35 °C for *A. baumannii*). Non-adherent cells were removed and the biofilms were gently rinsed twice with sterile phosphate-buffered saline (PBS) (Sigma-Aldrich, Merck Life Science S.r.l., Milan, Italy). The plates were air-dried under a laminar flow hood for 10 min to facilitate fixation of the sessile cells and then removed after 15 min. the dried sessile cells were then stained with 200 µL of a 2% (*w*/*v*) crystal violet (CV) solution per well for 20 min. The staining solution was discarded, and the plates were gently washed with sterile PBS. The retained crystal violet was subsequently solubilized with 200 μL of 20% (*w*/*v*) glacial acetic acid (Carlo Erba Reagents S.r.l., Milan, Italy) and the absorbance was recorded at 540 nm using a Cary Varian spectrophotometer (Cary Varian, Palo Alto, CA, USA). Biofilm inhibition was expressed as the percentage reduction compared to the untreated control, which was considered to be 0% inhibition. All experiments were performed in triplicate, and the results are presented as mean values. Although biofilm developmental kinetics may differ among bacterial species and even among strains, all microorganisms were incubated for 24 h under identical experimental conditions to obtain established biofilms suitable for comparative antibiofilm screening. The use of a standardized 24 h static microtiter plate model is widely accepted for the quantitative evaluation of bacterial biofilms and for screening the activity of antimicrobial compounds [94,95].

#### 4.8.4. Effects on Cell Metabolic Activity Within Biofilm

The 3-(4,5-dimethylthiazol-2-yl)-2,5-diphenyltetrazolium bromide (MTT) colorimetric method was employed to evaluate the effect of the EO on the metabolic activity of bacterial cells within the 24 h preformed biofilm [93]. Two concentrations of the EO, previously dissolved in sterile DMSO (to achieve final concentrations of 5 and 10 µg/mL), were added to each well. After the initial 24 h incubation required for biofilm formation, planktonic cells were removed and the treatment protocol described previously was applied. Following a further 24 h incubation, 150 µL of PBS and 30 µL of 0.3% MTT solution were added to each well. The microplates were incubated for 2 h at 37 °C (35 °C for *A. baumannii*) before the MTT solution was removed. Wells were then washed twice with 200 µL of sterile physiological solution, and 200 µL of DMSO were added to dissolve the formazan crystals. As for the CV test, although biofilm developmental kinetics differ among bacterial species and even among strains, all microorganisms were incubated for 24 h under identical experimental conditions to obtain established biofilms suitable for comparative antibiofilm screening. This incubation time is widely adopted in static microtiter plate assays and enables the evaluation of antimicrobial activity under standardized conditions, although species-specific differences in biofilm maturation cannot be completely excluded. Absorbance was measured at 570 nm (Cary Varian, Palo Alto, CA, USA). Experiments were performed in triplicate, and mean values were calculated.

### 4.9. Statistical Analysis

Statistical analysis of phytotoxic activity was performed by analysis of variance (ANOVA) using GraphPad Prism 6.0 (Software Inc., San Diego, CA, USA). The results were compared to the untreated control and considered statistically significant, by Dunnett’ s test, when *p* < 0.05.

Statistical analysis was performed separately for each bacterial strain and for each assay (CV and MTT). For each strain, untreated control and EO-treated samples were compared by one-way analysis of variance (ANOVA), followed by Tukey’s multiple comparison test. Differences were considered statistically significant at *p* < 0.05.

## 5. Conclusions

The EO obtained from horticultural by-products of *A. dracunculus* showed a broad spectrum of biological activities. It was able to inhibit root elongation of some selective weed species, an effect that may be associated with its enzymatic inhibitory capacity towards key enzymes involved in seed germination processes, while the moderate antioxidant activity may represent an additional but limited contributing factor. More importantly, the EO showed pronounced antibacterial and antibiofilm activity, proving particularly effective in reducing the metabolic activity of mature cells associated with biofilms. Although estragole has been identified as the main component of the EO and may contribute substantially to the observed biological effects, the highlighted activities should be considered as the result of the total phytochemical composition of the EO, where synergistic and/or additive interactions between the various components may contribute to manifest the final effects. Furthermore, the EO maintained a chemical profile comparable to that commonly reported for EOs obtained from conventional plant material. To further explore the valorization potential of *A. dracunculus* processing waste, future studies could focus on alternative extraction strategies, such as Soxhlet or ultrasound-assisted ethanol extraction. This would enrich the available information on the recovery of bioactive compounds from this biomass residue. Overall, the results suggest that the waste biomass generated during the processing of *A. dracunculus* represents a valuable source of bioactive compounds that can be effectively valorized within the principles of the circular economy, offering promising opportunities for the development of sustainable natural products for agri-food applications.

## Figures and Tables

**Table 1:** Composition of the essential oil.

N.	Compound	Content (%)	Ki ^a^	Ki ^b^	Identification ^c^
**1**	α-Pinene	1.13	932	1012	1,2,3
**2**	β-Pinene	0.14	971	1111	1,2,3
**3**	β-Myrcene	0.11	991	1167	1,2
**4**	Limonene	3.26	1022	1180	1,2,3
**5**	*cis*-β-ocimene	9.89	1038	1225	1,2
**6**	*trans*-β-ocimene	12.27	1048	1241	1,2
**7**	Linalool	0.1	1099	1581	1,2,3
**8**	*allo*-Ocimene	0.62	1124	1382	1,2
**9**	Estragole	70.17	1197	1671	1,2,3
**10**	Eugenol	0.89	1352	2156	1,2,3
**11**	β-Elemene	0.2	1387	1593	1,2
**12**	Methyleugenol	0.21	1400	2023	1,2
**13**	β-Caryophyllene	0.43	1406	1607	1,2
**14**	Germacrene D	0.15	1472	1708	1,2
**15**	Bicyclogermacrene	0.29	1489	1756	1,2
**16**	Caryophyllene epoxide	0.14	1573	1989	1,2
	Total	100.00			
	Hydrocarbon monoterpenes	27.42			
	Oxygenated monoterpenes	0.10			
	Hydrocarbon sesquiterpenes	1.07			
	Oxygenated sesquiterpenes	0.14			
	Phenylpropanoids	71.27			

^a, b^ The Kovats retention indices are relative to a series of *n*-alkanes (C10–C35) on the apolar HP-5MS and the polar HP Innowax capillary columns, respectively. ^c^ Identification method: 1 = comparison of the Kovats retention indices with published data, 2 = comparison of mass spectra with those listed in the NIST 17 and Wiley 275 libraries and with published data, and 3 = co-injection with authentic compounds.

**Table 2:** Antioxidant activity of the essential oil.

	DPPH IC_50_^1^ (mg/mL) (Mean ± SD) ^2^	FRAP μmol Fe^2+^ Equivalents/g EO (Mean ± SD)	ABTS TEAC ^3^ (μmol TE/g EO) (Mean ± SD)
*A. dracunculus* EO	2.15 ± 0.05	305.23 ± 10.56	396.42 ± 11.71
Trolox	(3.21 ± 0.21) × 10^−3^	5401.30 ± 952.31	/
Ascorbic acid	/	/	5746.11 ± 870.42

^1^ IC_50_ = concentration required to reduce the absorbance of DPPH by 50%. ^2^ Mean ± SD = indicates the mean value of the three experiments and the value of the standard deviation. ^3^ TEAC= Trolox equivalents antioxidant capacity.

**Table 3:** Enzymatic inhibitory activity of the essential oil.

	α-Amylase Inhibitory Activity IC_50_^1^ (mg/mL) (Mean ± SD) ^2^	α-Glucosidase Inhibitory Activity IC_50_ (mg/mL) (Mean ± SD)
*A. dracunculus* EO	1.53 ± 0.21	7.34 ± 0.69
Acarbose	(1.32 ± 0.11) × 10^−3^	(876.54 ± 48.22) × 10^−3^

^1^ IC_50_ = concentration required to inhibit 50% of the enzyme activity under the experimental conditions. ^2^ Mean ± SD = indicates the mean value of the three experiments and the value of the standard deviation.

**Table 4:** Phytotoxic activity of the essential oil.

Number of Germinated Seeds				
	***Hordeum vulgare***	***Raphanus sativus***	***Sinapis alba***	***Lolium multiflorum***
Control	0.00	0.00	0.00	0.00
Treatment (µg/mL)				
125	−4.13	8.71	3.52	13.64
250	20.88	−8.6	0	−13.64
500	4.13	−26.08	3.52	0
**Radical length (cm)**				
	***Hordeum vulgare***	***Raphanus sativus***	***Sinapis alba***	***Lolium multiflorum***
Control	0.00	0.00	0.00	0.00
Treatment (µg/mL)				
125	30.32	37.39	11.5	−12.36
250	−7.74	28.89	15.5	−5.26
500	3.23	14.73	44.5	−5.72

Control: Mixture of water:acetone (99.5:0.5 *v*/*v*).

**Table 5:** Inhibition of 24 h preformed biofilm biomass determined by CV assay.

Strain	2 µL/mL	4 µL/mL	6 µL/mL
*A. baumannii*	37.87 ± 3.34 ^b^	43.52 ± 4.58 ^b^	45.00 ± 2.36 ^b^
*E. coli*	15.23 ± 1.35 ^b^	23.01 ± 2.09 ^bc^	34.47 ± 2.19 ^c^
*K. pneumoniae*	21.16 ± 7.80 ^b^	22.08 ± 1.49 ^b^	22.56 ± 1.32 ^b^
*L. monocytogenes*	1.19 ± 0.06 ^a^	20.74 ± 1.42 ^b^	29.39 ± 1.28 ^c^
*P. aeruginosa*	35.70 ± 2.47 ^b^	37.86 ± 2.59 ^b^	51.25 ± 2.13 ^c^
*S. aureus*	20.02 ± 2.30 ^ab^	33.24 ± 2.66 ^b^	41.04 ± 1.47 ^b^

Values represent the percentage reduction of biofilm biomass relative to the untreated control of the corresponding bacterial strain. Data are expressed as mean ± SD of three independent experiments. Different superscript letters within the same row indicate statistically significant differences among EO concentrations according to one-way ANOVA followed by Tukey’s multiple comparison test (*p* < 0.05).

**Table 6:** Inhibition of sessile-cell metabolic activity determined by MTT assay.

Strain	2 µL/mL	4 µL/mL	6 µL/mL
*A. baumannii*	30.66 ± 3.14 ^a^	53.45 ± 1.27 ^b^	55.23 ± 1.54 ^b^
*E. coli*	10.15 ± 1.03 ^a^	51.98 ± 0.50 ^b^	75.79 ± 1.25 ^c^
*K. pneumoniae*	29.54 ± 2.71 ^a^	55.85 ± 0.05 ^ab^	69.28 ± 1.62 ^b^
*L. monocytogenes*	40.00 ± 2.92 ^a^	61.42 ± 1.02 ^b^	79.91 ± 3.87 ^c^
*P. aeruginosa*	10.12 ± 0.44 ^a^	40.55 ± 2.72 ^b^	75.40 ± 3.67 ^c^
*S. aureus*	16.64 ± 0.45 ^a^	29.16 ± 1.90 ^b^	74.80 ± 4.36 ^c^

Values represent the percentage reduction of biofilm biomass relative to the untreated control of the corresponding bacterial strain. Data are expressed as mean ± SD of three independent experiments. Different superscript letters within the same row indicate statistically significant differences among EO concentrations according to one-way ANOVA followed by Tukey’s multiple comparison test (*p* < 0.05).

## Data Availability

The original contributions presented in this study are included in the article. Further inquiries can be directed to the corresponding authors.
